# Radiomic analysis reveals diverse prognostic and molecular insights into the response of breast cancer to neoadjuvant chemotherapy: a multicohort study

**DOI:** 10.1186/s12967-024-05487-y

**Published:** 2024-07-08

**Authors:** Ming Fan, Kailang Wang, Da Pan, Xuan Cao, Zhihao Li, Songlin He, Sangma Xie, Chao You, Yajia Gu, Lihua Li

**Affiliations:** 1 Institute of Biomedical Engineering and Instrumentation, Hangzhou Dianzi University, Xiasha High Education Zone, Hangzhou, 310018 Zhejiang China; 2 Department of Radiology, Fudan University Shanghai Cancer Center, Shanghai, China

**Keywords:** Radiomics analysis, Shrinkage pattern, Multitask learning, Prognosis

## Abstract

**Background:**

Breast cancer patients exhibit various response patterns to neoadjuvant chemotherapy (NAC). However, it is uncertain whether diverse tumor response patterns to NAC in breast cancer patients can predict survival outcomes. We aimed to develop and validate radiomic signatures indicative of tumor shrinkage and therapeutic response for improved survival analysis.

**Methods:**

This retrospective, multicohort study included three datasets. The development dataset, consisting of preoperative and early NAC DCE-MRI data from 255 patients, was used to create an imaging signature-based multitask model for predicting tumor shrinkage patterns and pathological complete response (pCR). Patients were categorized as pCR, nonpCR with concentric shrinkage (CS), or nonpCR with non-CS, with prediction performance measured by the area under the curve (AUC). The prognostic validation dataset (n = 174) was used to assess the prognostic value of the imaging signatures for overall survival (OS) and recurrence-free survival (RFS) using a multivariate Cox model. The gene expression data (genomic validation dataset, n = 112) were analyzed to determine the biological basis of the response patterns.

**Results:**

The multitask learning model, utilizing 17 radiomic signatures, achieved AUCs of 0.886 for predicting tumor shrinkage and 0.760 for predicting pCR. Patients who achieved pCR had the best survival outcomes, while nonpCR patients with a CS pattern had better survival than non-CS patients did, with significant differences in OS and RFS (p = 0.00012 and p = 0.00063, respectively). Gene expression analysis highlighted the involvement of the IL-17 and estrogen signaling pathways in response variability.

**Conclusions:**

Radiomic signatures effectively predict NAC response patterns in breast cancer patients and are associated with specific survival outcomes. The CS pattern in nonpCR patients indicates better survival.

**Supplementary Information:**

The online version contains supplementary material available at 10.1186/s12967-024-05487-y.

## Introduction

Neoadjuvant chemotherapy (NAC) is a fundamental component of treatment for early-stage breast cancer. Achieving a pathological complete response (pCR) after NAC has been consistently linked with superior long-term clinical outcomes, as evidenced by numerous studies [1–3]. In addition to serving as a treatment endpoint, early assessment during NAC is crucial for guiding clinical decisions and tailoring treatment strategies [4]. NAC generally involves several cycles, leading to various patterns of tumor shrinkage. Among these, concentric shrinkage (CS) is associated with a better prognosis and, in addition to pCR, may serve as an independent predictive factor for patient outcomes [5]. Nonetheless, the relationship between specific response patterns and prognosis, particularly among patients who do not achieve a pCR, is still not well understood. Elucidating the heterogeneity of these responses is essential for evaluating their prognostic value and improving treatment approaches for breast cancer patients.

Dynamic contrast-enhanced magnetic resonance imaging (DCE-MRI) is a highly specific and sensitive [6, 7] for effectively predicting NAC responses in patients with breast cancer [8–10]. Radiogenomic analysis based on preoperative DCE-MRI has revealed imaging signatures associated with recurrence in estrogen receptor-positive breast cancer patients, and these signatures can serve as prognostic biomarkers for survival and NAC responsiveness [11]. Recent research has developed a prognostic model that combines radiomic data from multiparametric MR images with clinicopathological factors to predict tumor regression in NAC-treated breast cancer patients [12]. Given these insights, preoperative DCE-MRI signatures constitute a comprehensive approach for predicting NAC response and tailoring personalized treatment plans for breast cancer patients.

In addition to initial preoperative evaluations, longitudinal imaging plays an integral role at several points during NAC to both monitor shrinkage patterns during NAC and predict treatment responses in breast cancer patients [13, 14]. By examining changes in tumor heterogeneity prior to and at the onset of NAC, DCE-MRI has proven effective in predicting therapeutic outcomes [15]. Studies have consistently shown that temporal changes in pharmacokinetic parameters, tumor dimensions, and MRI texture features correlate with breast cancer patients’ responses to NAC [16, 17].

Despite these advances, the relationship between tumor response patterns and patient prognosis has not been fully explored. Although achieving a pCR is widely accepted as a favorable prognostic indicator, the clinical outcomes of patients who exhibit diverse patterns of tumor regression without achieving a pCR have not been fully delineated. Our study aimed to shed light on the prognostic significance of various tumor response patterns and their biological underpinnings, particularly in the context of pCR and the dynamic changes observed during therapy. By integrating radiological evaluations with an analysis of response kinetics, we endeavored to enhance the precision of survival prognostications for patients with breast cancer.

## Materials and methods

### Dataset

This study received approval from the Institutional Review Board, and informed consent was obtained from all participants. We consecutively included women who underwent NAC and preoperative MRI. The development dataset included 259 patients who received NAC. All patients underwent the standard NAC protocol, which included six to eight cycles of the taxotere–epirubicin–cyclophosphamide (TEC) regimen. The entire NACT course lasted approximately 4 to 5 months, with each cycle administered at intervals of approximately 20 days. This dataset included longitudinal imaging data, including preoperative and early NAC DCE-MRI data, which facilitated the construction of a model to assess tumor shrinkage patterns during early NAC.

For prognostic validation, we used a separate dataset of women from the ISPY1 trial available in The Cancer Imaging Archive (TCIA) [18]. This dataset contains DCE-MRI, clinical and follow-up information, which serves to validate the prognostic relevance of the imaging biomarkers. To interpret the molecular context of the imaging features, the genomic validation dataset utilized in this study consisted of MRI data from the prognostic validation dataset, while the gene expression data were sourced from the GSE32063 and GPL14668 datasets [19].

**Fig. 1:**
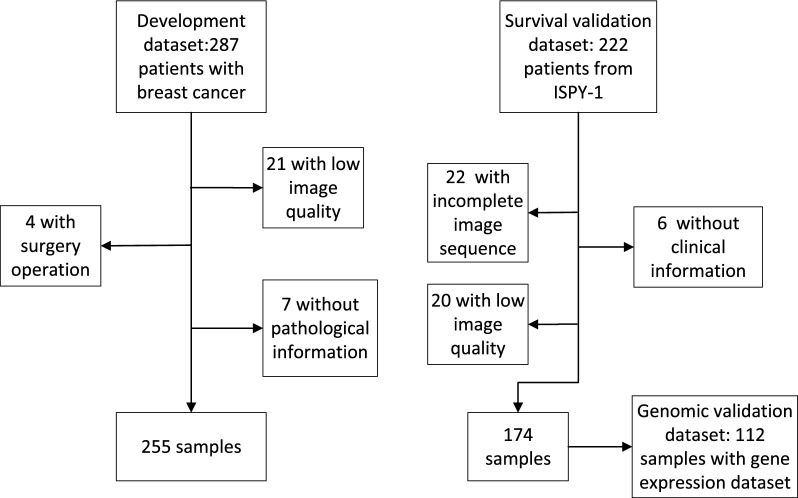
Data overview

The exclusion criteria were as follows: patients lacking preoperative DCE-MRI or incomplete DCE-MRI data, patients missing pathology reports, patients whose tumors were not clearly visible or could not be confidently described or annotated by an experienced radiologist, and patients with diffuse tumors. The data collection framework is depicted in Fig. 1.

### Framework overview

**Fig. 2:**
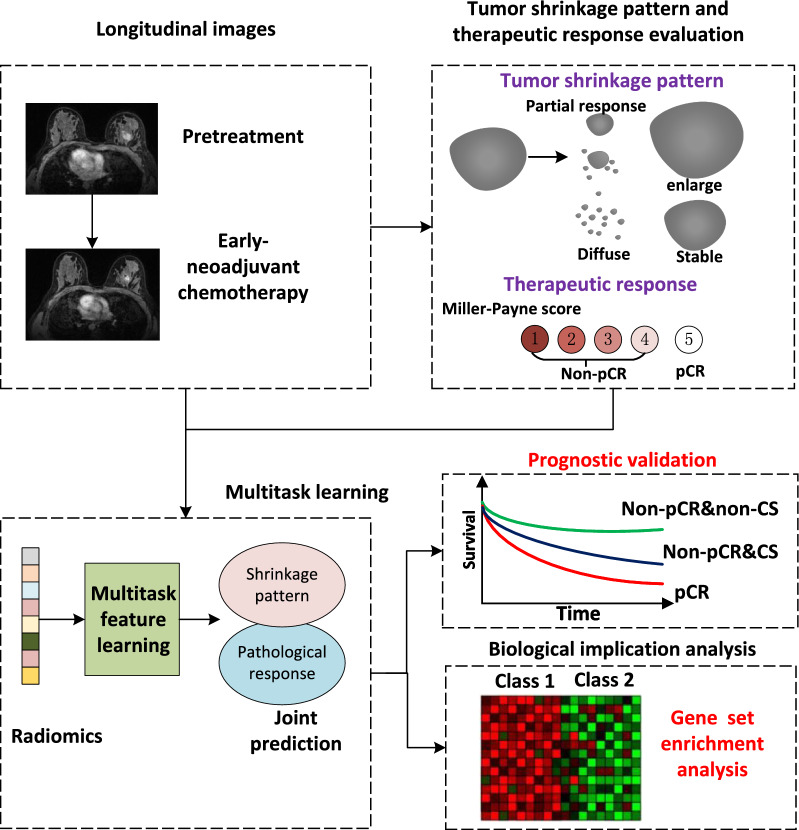
Framework overview

Figure 2 shows an overview of the framework. In the development dataset, multitask learning was implemented to jointly predict pCR and shrinkage status in breast cancer patients. The dataset was randomly partitioned into a training set, constituting 66% of the samples for model construction, and an inner validation set, comprising the remaining 33%, was used for model validation. The imaging features and trained model parameters of the patients in the development dataset were applied to the DCE-MRI data in the prognostic validation set to simultaneously predict the likelihood of CS and pCR following NAC. Patients were stratified into three categories according to the predicted response patterns: Group 1 (nonpCR and non-CS), Group 2 (nonpCR and CS), and Group 3 (pCR). We assessed survival differences among the three groups with distinct response patterns. Furthermore, the biological relevance of the differentially expressed genes (DEGs) identified across these groups was elucidated to reveal the molecular implications underlying the response patterns.

### Imaging protocol

For the development dataset, DCE-MRIs were acquired using a Siemens 3.0 T scanner in the prone position. This involved capturing a precontrast image and a series of five postcontrast images. The protocol specified a TR of 4.5 ms, a TE of 1.56 ms, a 10° flip angle, a field of view of 384 × 384, a 2.2 mm slice thickness, and an in-plane resolution of 0.9375 mm. The time intervals between the subsequent postcontrast phases were 60 s.

For prognostic evaluation, data were collected from I-SPY1 in the TCIA, and a detailed imaging protocol has been described elsewhere [20]. DCE-MR images were acquired using a Siemens or GE 1.5 scan system. The imaging protocol was as follows: TR, 20 ms or less; TE, 4.5 ms; flip angle, 45° or less; field of view, ranging from 16 to 18 mm; and a 256 × 192 matrix. Each image consisted of up to 64 slices, each no more than 2.5 mm thick, and an in-plane resolution not exceeding 1 mm. Following contrast agent injection, early postcontrast series were acquired at approximately 2-min intervals. The patient data included in both datasets were breast sagittal plane images.

### Image preprocessing

The DCE-MRI images were initially subjected to a standardization process to ensure uniform spatial resolution across the dataset, with all images being resampled to a consistent voxel size of 0.8 mm. Intensity normalization was performed to reduce variations in the differing imaging protocols. This was achieved by scaling the grayscale values of the images such that the normalization factor was the mean intensity derived from the interquartile range (IQR), specifically between the upper and lower quartiles of the grayscale intensity distribution. Subsequently, the tumor regions of interest (ROIs) were identified on the intermediate postcontrast series using a spatial fuzzy C-means (FCM) algorithm. The parenchymal area, inclusive of a peripheral margin approximately 20 mm wide encircling the tumor, was segmented for feature analysis. Image features were extracted from three distinct sequences: the precontrast image (S0), the intermediate postcontrast image (SM), and the subtraction image (SM0). The SM, typically captured approximately 2 min following the injection of the contrast agent, represents the phase of peak enhancement in the time-series MRI. SM0 was generated by subtracting S0 from SM, thereby enhancing the visualization of contrast agent accumulation.

### Histopathological analysis

Experienced pathologists with more than 15 years of professional experience who were blinded to the MRI results performed comprehensive histopathological analyses on full-face sections of breast tissue specimens. The Elston–Ellis modification of the Scarff–Bloom–Richardson grading system [21] was used to determine the histological grade of the tumors. Based on the recommendations of the St. Gallen International Breast Cancer Conference [22], a Ki-67 labeling index exceeding 14% was considered positive, whereas the other indices were considered negative. Histological grades 1 and 2 were amalgamated into a low-grade category, whereas grade 3 was indicative of a high-grade malignancy. Immunohistochemical examination of estrogen receptor (ER), progesterone receptor (PR), and human epidermal growth factor receptor 2 (HER2) status was performed via the streptavidin–peroxidase method [23]. The luminal A breast cancer subtype was characterized by the coexpression of ER and PR with a concurrent absence of HER2 overexpression or amplification. Conversely, the luminal B subtype was distinguished by the presence of ER or PR combined with either HER2 overexpression/amplification or a high Ki-67 proliferation index in the absence of HER2 overexpression.

### Feature extraction

Tumor feature extraction was performed using precontrast (S0) imaging sequences, middle enhancement (SM) sequences, and subtraction of the middle enhancement sequence and the S0 sequence (SM0). A total of 102 radiomic features were extracted from each image sequence or map utilizing the open-source software package PyRadiomics [24]. The range of extracted features encompassed diverse categories, which included morphological features (n = 14), first-order statistical features (n = 18), and texture features (n = 70). The texture features included features based on the gray level co-occurrence matrix (GLCM) (n = 24), the gray-level run-length matrix (GLRLM) (n = 16), the gray-level size-zone matrix (GLSZM) (n = 16), and those rooted in the gray-level spatial dependence matrix (GLDM) (n = 14).

### Assessment of therapeutic response

The response to NAC was assessed using the Miller–Payne (MP) grading system. A score of 5 (pCR) indicated the responsive group, while scores ranging from 1 to 4 indicated the nonresponsive group. pCR was defined as no invasive residual tumor in the breast or nodes or only residual ductal carcinoma in situ. Tumor shrinkage patterns were categorized into two main types after receiving NAC: CS and non-CS. The CS pattern consisted of three subtypes: pCR, simple CS, and CS with focal enhancements. Simple CS was characterized by a reduction in the maximum tumor diameter without surrounding enhancement and was observed only in tumors with prior enhancement. A CS with focal enhancement was defined as a primary mass enhancement lesion exhibiting a CS pattern, albeit with the presence of residual foci. In contrast, non-CSs were divided into four subtypes: diffuse decrease, indicating a widespread reduction in tumor size; enlarged tumor, where the lesion increased in size; intensity decrease, where the lesion showed a reduction in density; and no change, where no alterations were observed.

### Survival analysis

Kaplan–Meier curves were used to compare survival among different patient groups. Univariate Cox proportional hazards regression models were used to analyze the relationships between imaging signatures and recurrence-free survival (RFS) and overall survival (OS) rates. The log-rank test was used to evaluate the significance of disparities observed among the survival curves; delineation of the cutoff point was established through identification of an optimal threshold, which corresponded to the minimum log-rank p value obtained. Multivariate Cox regression analysis was conducted to assess the independent correlation between the imaging signatures and OS or RFS, controlling for clinical variables, including age, ER status, PR status, HER2 status, and tumor size. Patients who remained event-free at the conclusion of the 10-year period were censored at this juncture. To determine the prognostic value of the imaging signatures, we applied a likelihood ratio test. The hazard ratio (HR) was employed as a measure in the survival analysis.

### Statistical analysis

To address the collinearity among the collective imaging features in the model, those with a Pearson correlation coefficient greater than 0.9 were excluded if they also demonstrated a high average correlation coefficient with other features.

Building upon our previous work [25], we used a multitask random forest model to identify a common feature subset relevant to all tasks. Initially, a random forest model was constructed using data from all tasks. Each tree in the forest was trained on a randomly sampled subset of the combined dataset, fostering diversity and robustness in our feature selection. During each node split, features were evaluated based on their collective impact on the accuracy of all tasks. This process resulted in a feature importance ranking that captured the relevance of each feature to all tasks simultaneously. We then selected the top-ranked features to form a common feature subset that yielded the best average performance on leave-one-out cross-validation (LOOCV) using the area under the receiver operating characteristic curve (AUC) as the model evaluation criterion. We employed a random grid search method to determine the hyperparameters of the random forest models. By incorporating this multitask feature selection approach, we were able to identify a set of features that were not only predictive of individual tasks but also captured the underlying commonalities between the tasks of predicting pCR and shrinkage patterns. Finally, we used the trained models for prediction on the testing set and calculated the AUC to evaluate the model performance.

The differences in the AUC between the models were evaluated using the bootstrap method. Additionally, analysis of variance (ANOVA) was used to assess the differences in gene expression between the groups.

### Genomic interpretability analysis

To explore the molecular foundations underlying patient variability in CS or pCR responses to treatment, we conducted a genomic analysis focused on gene function. We excluded genes that were expressed in only 20% of patients as well as those lacking expression values. Furthermore, we narrowed our focus by eliminating genes with minimal variance in their expression patterns across patients, ultimately selecting the top 2000 genes with the greatest variance. This approach ensured that only the most significant genetic features were retained for subsequent analysis. The associations of these genes with response status were subsequently analyzed (i.e., pCR, CS and nonpCR, non-CS and nonpCR). Furthermore, genes that demonstrated a significant association were analyzed using gene set enrichment analysis (GSEA) with reference to the Kyoto Encyclopedia of Genes and Genomes (KEGG) to assess the influence of particular pathways on the response categories. To assess the correlation between gene expression and treatment response or tumor shrinkage patterns, we employed a regression model. Our comprehensive approach provides a detailed understanding of how genetic factors correlate with patient treatment responses.

## Results

### Patient characteristics

Table 1 presents the clinical characteristics of the patients from the two datasets, which included age, family history, menopausal status, maximum tumor diameter, hormone receptor status, HER2 status, recurrence, death, the MP score, and tumor shrinkage patterns. The development dataset included 255 female patients ranging in age from 25.0 to 79.0 years, with a mean age of 48.9 ± 10.4 years (standard deviation). The prognostic validation dataset included 174 female patients aged between 26.7 and 68.8 years, with a mean age of 47.8 ± 8.7 years (standard deviation). The genomic validation dataset included a gene expression set (n = 112).

**Table 1:** Patient characteristics

Characteristics	Development dataset (n = 255)	Prognostic validation dataset (n = 174)
Age (y)		
Range	27.0–79.0	26.7–68.8
Median	49.0	48.2
Mean ± std	48.9 ± 10.4	47.8 ± 8.7
Family history		
No	194 (76.1%)	N/A
Yes	57 (22.4%)	N/A
Unknown	4 (1.5%)	N/A
Menopausal status		
Pre	104 (40.8%)	N/A
Post	144 (56.5%)	N/A
Unknown	7 (2.7%)	N/A
Maximum tumor diameter (cm)		
Range	10–92	19–184
Median	35	68
Mean ± std	37.6 ± 17.0	68.5 ± 31.1
Progesterone receptor		
Positive	117 (45.9%)	82 (47.1%)
Negative	138 (54.1%)	92 (52.9%)
Estrogen receptor		
Positive	149 (58.4%)	100 (57.5%)
Negative	106 (41.6%)	74 (42.5%)
Human epidermal growth factor receptor 2		
Positive	111 (43.5%)	51 (29.3%)
Negative	144 (56.5%)	123 (70.7%)
Recurrence	N/A	
Event	N/A	46 (57.5%)
No event	N/A	128 (42.5%)
Death	N/A	
Event	N/A	30 (17.2%)
No event	N/A	144 (82.8%)
Miller–Payne score		
Nonresponse (1–4)	187 (73.3%)	N/A
Response (5)	68 (26.7%)	N/A
Pattern of tumor shrinkage		
Non-CS	58 (22.7%)	N/A
CS	197 (77.3%)	N/A

### Multitask model for jointly predicting pCR and shrinkage patterns in breast cancer patients

Table 2 provides a comparative analysis of the performance of single-task learning models and multitask learning models in predicting response patterns to NAC. A detailed illustration of the imaging signatures (n = 17) used is provided in Table S1. The findings indicate that multitask learning models outperform their single-task counterparts in predicting both CS and pCR. Specifically, the multitask learning model demonstrated superior prediction accuracy, achieving an AUC of 0.886 for CS and 0.760 for pCR, in contrast to the single-task learning models, which exhibited AUCs of 0.876 for CS and 0.753 for pCR.

**Table 2:** Performance for single-task and multitask prediction of pathological response and shrinkage patterns

	AUC (95% CI)	Sensitivity	Specificity	Precision	F1
CS-multitask	0.886 (0.817–0.957)	0.836	0.800	0.933	0.882
CS-single task	0.876 (0.796–0.956)	0.731	0.900	0.961	0.831
pCR multitask	0.760 (0.652–0.871)	0.826	0.656	0.463	0.594
pCR single task	0.753 (0.651–0.856)	1	0.438	0.390	0.561

**Fig. 3:**
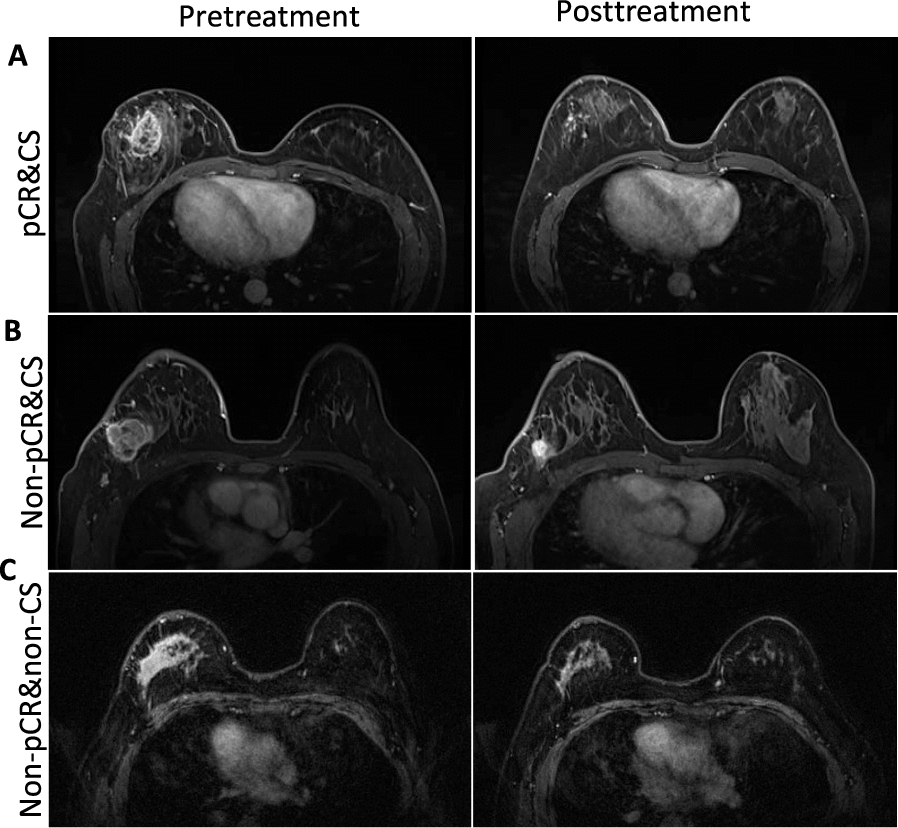
Illustrative cases depicting diverse tumor shrinkage and pathologic response patterns. **A** A 33-year-old patient with breast cancer who achieved a pathological complete response (pCR) and concentric shrinkage (CS) following neoadjuvant chemotherapy (NAC). The sphericity values pre- and posttreatment were 0.438 and 0.532, respectively. **B** A 52-year-old breast cancer patient who did not achieve pCR yet demonstrated CS after NAC. The pre- and early-NAC sphericity values were 0.487 and 0.457, respectively. **C** A 46-year-old patient with breast cancer who neither achieved pCR nor exhibited CS following NAC. The sphericity values recorded before and after treatment were 0.365 and 0.766, respectively

Figure 3 shows the individual imaging feature values and sphericities for stratifying patients into specific response patterns. Tumor sphericity increased from 0.438 in the pretreatment image to 0.532 in the early NAC image, which was correlated with patients who achieved a pCR. In contrast, patients who did not achieve a pCR but did achieve a CS exhibited a reduction in tumor sphericity from 0.487 to 0.457. Remarkably, for patients who were nonpCR or non-CS, a substantial increase in tumor sphericity was observed, with the value increasing from 0.365 in the preoperative images to 0.766 in the early-NAC images.

### Validation of the imaging signatures of shrinkage pattern and pathological complete response for survival prediction

**Fig. 4:**
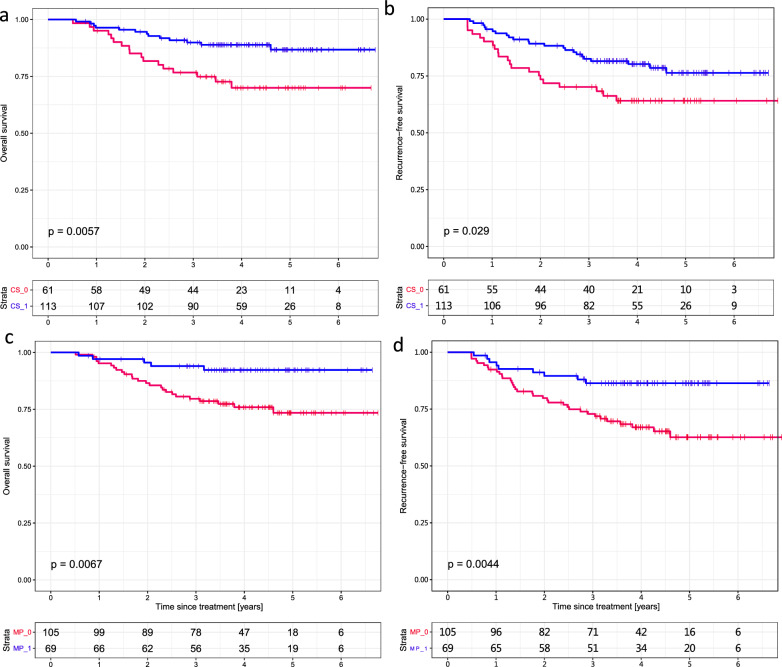
Validation of the imaging signature-predicted shrinkage patterns and pathological complete response status for survival analysis. The predicted concentric shrinkage (CS) was used to evaluate **a** overall survival (OS) and **b** recurrence-free survival (RFS). The pCR labels were validated for **c** OS and **d** RFS

The clinical significance of the imaging signatures was further validated through survival analysis using a prognostic validation dataset to assess the impact of shrinkage pattern status on breast cancer prognosis (Fig. 4 and Table S2). A predicted CS showed a positive correlation (threshold value of 0.6708) with improved OS (p = 0.0057) and RFS (p = 0.029) (Fig. 4a, b, respectively). Moreover, positive correlations were found between imaging-predicted pCR and improved OS (p = 0.0067) and RFS (p = 0.0044) (Fig. 4c, d, respectively) under a threshold of 0.2444. Overall, the predicted tumor shrinkage pattern demonstrated marginally superior prognostic performance compared to the predicted pCR status, as evidenced by more statistically significant p values in the prognostic model.

### Survival validation of the imaging signature-generated response pattern

**Fig. 5:**
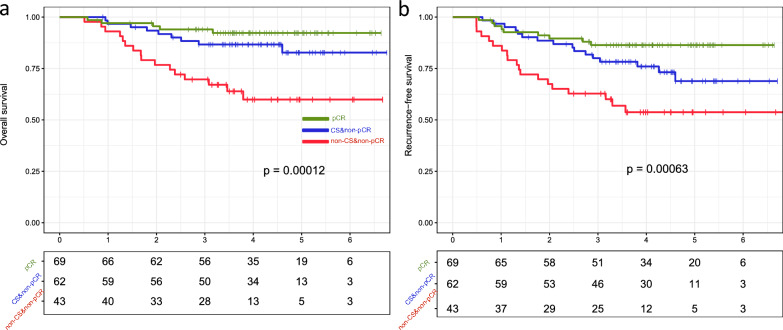
Survival outcome prognostic validation of imaging-based response patterns. Among patients who had a nonpathologic complete response (nonpCR), those exhibiting concentric shrinkage (CS) demonstrated improved **a** overall survival (OS) and **b** recurrence-free survival (RFS) compared to patients with non-CS patterns

Patients were separated into three groups based on the imaging signature-generated CS and pCR status (i.e., pCR, nonpCR and CS, and nonpCR and non-CS). Among these groups, significant differences in OS and RFS were observed, with p values of 0.00012 and 0.00063, respectively. As anticipated, patients who achieved pCR had more favorable survival outcomes than did those who did not. Moreover, within the nonpCR population, individuals exhibiting a CS pattern had improved survival compared to those exhibiting a non-CS pattern (Fig. 5a, b).

### Image feature analysis for predicting survival in patients with breast cancer

The findings revealed that the predicted CS label was independently associated with improved RFS (HR = 0.539; 95% CI = 0.297–0.978; P = 0.042) and OS (HR = 0.426; 95% CI = 0.204–0.887; P = 0.023) after controlling for clinical information (Table 3). Similarly, the predicted pCR also emerged as an independent prognostic factor for RFS (HR = 0.473; 95% CI = 0.230–0.971; P = 0.041) and OS (HR = 0.339; 95% CI = 0.128–0.896; P = 0.029) when accounting for clinical and pathological features. These findings reinforce the clinical relevance of tumor response patterns in determining the prognostic trajectories of patients post-NAC.

**Table 3:** Multivariate Cox regression analysis of the associations of the predicted response patterns and clinicopathological factors with survival outcomes

Category	Recurrence free survival		Overall survival	
	HR	p	HR	p
Shrinkage pattern				
PR	0.607 (0.328–1.121)	0.111	0.474 (0.214–1.047)	0.065
HER2	1.228 (0.650–2.318)	0.527	1.202 (0.559–2.583)	0.637
Age	0.978 (0.943–1.015)	0.244	1.004 (0.960–1.050)	0.875
Largest diameter	1.008 (1.000–1.017)	0.062	1.009 (0.998–1.020)	0.097
Predicted CS	0.539 (0.297–0.978)	0.042	0.426 (0.204–0.887)	0.023
Pathological response				
PR	0.669 (0.362–1.235)	0.198	0.486 (0.220–1.073)	0.074
HER2	1.344 (0.719–2.513)	0.354	1.346 (0.638–2.840)	0.436
Age	0.979 (0.945–1.016)	0.263	1.005 (0.961–1.051)	0.834
Largest diameter	1.007 (0.998–1.015)	0.112	1.008 (0.998–1.019)	0.129
Predicted pCR	0.473 (0.230–0.971)	0.041	0.339 (0.128–0.896)	0.029

### Analysis of consistency in image features

As shown in Table S3, univariate Cox regression analysis revealed that imaging features in the multitask learning model were significantly associated with CS and pCR. For example, the sphericity feature—which reflects a tumor’s tendency toward a spherical conformation—was more frequently observed in CS-classified tumors and was positively correlated with a greater likelihood of pCR.

**Fig. 6:**
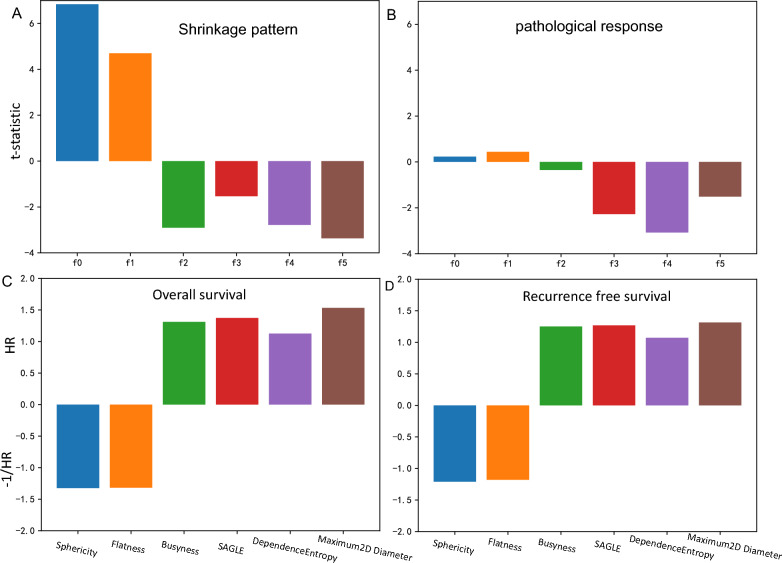
Bar plots illustrating imaging feature analysis for **a** tumor shrinkage pattern evaluation, **b** pathological response prediction, **c** overall survival, and **d** recurrence-free survival. The analyzed features included sphericity and flatness, busyness from the parenchymal region in intermediate postcontrast images, small areas with high gray-level emphasis from the lesion region in precontrast images, dependence entropy from the lesion region in precontrast images, and a maximum 2D diameter slice from the parenchymal region in intermediate postcontrast images. Inverse hazard ratios (HRs) are presented as − 1/HR for values less than one. *SAHGLE* small area high gray level emphasis

Six representative features that significantly contributed to the prediction of either CS status or pCR are shown in Fig. 6; these included flatness, small area high gray-level emphasis, and dependence entropy derived from the tumor region, as well as busyness and long-run low gray-level emphasis features extracted from the parenchymal region (Fig. 6). Higher values of sphericity or flatness are associated with CS and good survival. Conversely, higher values of busyness, small area high gray-level emphasis, dependence variance, and maximum diameter were associated with non-CSs or nonpCRs and poor survival outcomes. These figures demonstrate the consistency of the features in reflecting tumor shrinkage patterns and in predicting pathological response.

**Fig. 7:**
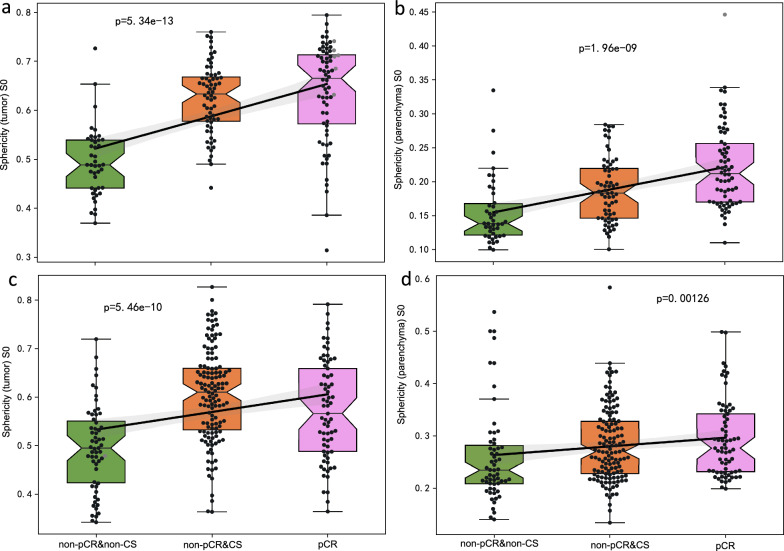
Sphericity features in the nonpCR and non-CS, nonpCR and CS, and pCR groups. Analysis of the sphericity features across **a** tumor and **b** parenchyma with a 20 mm width surrounding a tumor in the development dataset, as well as **c** tumor and **d** parenchyma with a 20 mm width surrounding a tumor in the validation dataset

We evaluated imaging features among three patient groups categorized by their different response patterns. These features demonstrated a trend toward either a decrease or increase in expression, corresponding to patient outcomes ranging from complete response (pCR) to nonpCR with non-CSs. For instance, sphericity in the tumor or parenchymal region correlated with these groups (Fig. 7). The greatest sphericity was observed in patients who achieved a pCR, with moderately lower sphericity in nonpCR patients with CS and the lowest sphericity in nonpCR patients without CS.

### Biological implications of imaging-based response patterns

By examining gene profiles corresponding to imaging-based response patterns in breast cancer, we identified 89 genes with significant prognostic correlations (p < 0.05). Subsequent pathway enrichment analysis revealed 12 pathways significantly associated with these genes (corrected p < 0.05) (Table 4). The IL-17 signaling pathway, which is involved in inflammation, was the most significantly enriched pathway (p = 2.88e−6). Elevated expression levels of genes within this pathway have been linked to poor prognosis in breast cancer patients [26, 27]. Moreover, the estrogen signaling pathway, another pathway of significance, has been associated with breast cancer metastasis [28]. This pattern aligns with the trends noted in the imaging signature analysis.

**Table 4:** Analysis of pathways related to marker genes across treatment response groups (non-CS, nonpCR, CS, and pCR)

Pathway	P value	Corrected p	Gene markers
IL-17 signaling pathway	2.88e−06	0.00041	S100A7|MMP13|S100A9|S100A8|MMP9
Estrogen signaling pathway	3e−4	0.0077	ATF6B|BCL2|KRT19|MMP9
Protein processing in endoplasmic reticulum	5.8e−4	0.012	OS9|ATF6B|EDEM2|BCL2
ECM-receptor interaction	0.0011	0.018	AGRN|LAMC3|ITGA6
Epstein–Barr virus infection	0.0012	0.020	OAS1|OAS3|HLA-G|BCL2
Small cell lung cancer	0.0013	0.021	LAMC3|ITGA6|BCL2
Parathyroid hormone synthesis, secretion and action	0.0019	0.026	ATF6B|MMP13|BCL2
Toxoplasmosis	0.0023	0.029	LAMC3|ITGA6|BCL2
Autophagy—animal	0.0032	0.035	WIPI1|DEPTOR|BCL2
Relaxin signaling pathway	0.0034	0.036	ATF6B|MMP13|MMP9
Dopaminergic synapse	0.0034	0.036	KIF5C|ATF6B|PPP1R1B
Measles	0.0040	0.040	OAS1|OAS3|BCL2

**Fig. 8:**
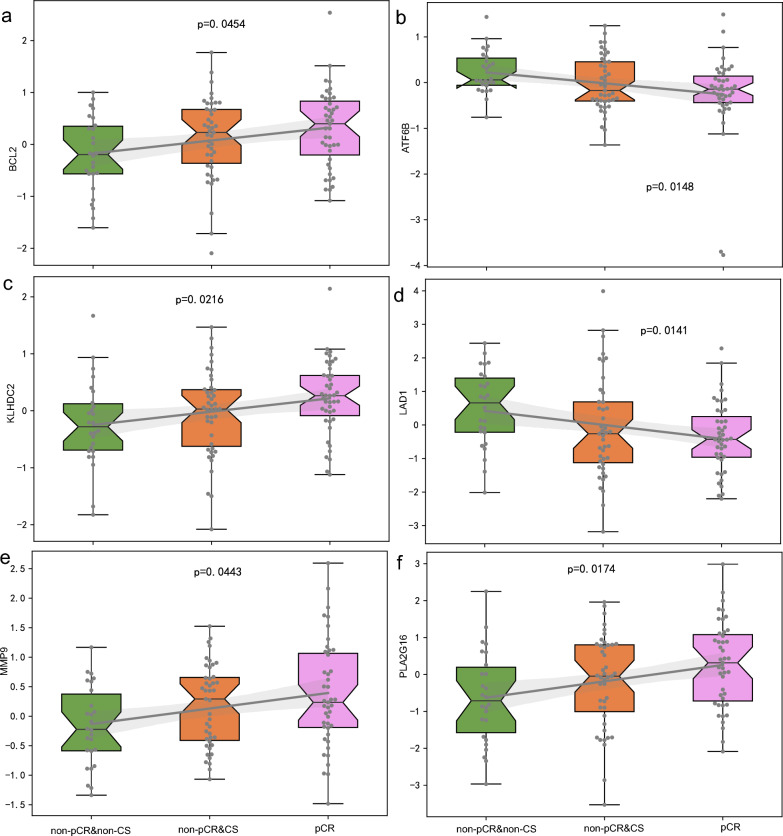
Expression profiles of key genes associated with breast cancer status. **A** BCL2; **B** ATF6B; **C** KLHDC2; **D** LAD1; **E** MMP9; **F** PLA2G16

As illustrated in Fig. 8, genes such as BCL2, KLHDC2, MMP9, and PLA2G16 showed a positive correlation with treatment response status. In contrast, ATF6B and LAD1 were negatively associated with this status. Specifically, increased expression of BCL2 was associated with a shift from pCR to non-CS or nonpCR, indicating a less favorable treatment response. These findings are consistent with those of a previous study in which negative BCL2 expression was significantly associated with pCR [29]. Moreover, increased expression of ATF6B has been correlated with a negative prognosis in patients with breast cancer [31], supporting our findings.

## Discussion

Our study employed noninvasive imaging to simultaneously predict tumor shrinkage and therapeutic response to NAC in breast cancer patients. We confirmed that patients who achieved a pCR had the best OS and RFS. Notably, among those unlikely to achieve pCR, patients with predicted CS patterns had improved survival compared to those without CS. This method allows early-stage prediction of CS patterns during NAC, providing valuable insights for patients who are unlikely to achieve pCR. Additionally, our genomic analysis revealed the significant presence of cancer-related pathways, including those related to IL-17 and estrogen signaling, among the genes whose expression varied significantly between the groups with distinct response groups. Overall, our research established a strong, independent link between imaging-based prognostic markers and survival in breast cancer patients.

In this study, we aimed to enhance the accuracy of predicting treatment responses by developing and validating a radiomic model that forecasts the patterns of breast cancer shrinkage during the initial two cycles of NAC. Previous studies have indicated that MRI-observed tumor response patterns at the midpoint of the NAC can predict pathological outcomes more accurately than posttreatment tumor response patterns [30]. Our model capitalizes on these early treatment stages to provide critical prognostic information. This early prediction model holds promise for optimizing therapeutic regimens and advancing patient care by enabling clinicians to make more informed and timely decisions about treatment efficacy.

In our analysis, we delineated gene signaling pathways correlated with distinct patterns of treatment response. Genes such as BCL2 and ATF6B are involved in these pathways. Previous studies have demonstrated that downregulation of BCL2 correlates with improved disease-free survival (DFS) in breast cancer patients [31]. Additionally, the upregulation of ATF6B expression has been linked to favorable DFS outcomes [32]. Additionally, elevated PLA2G16 gene expression has been correlated with improved DFS, consistent with findings in the literature [33]. Our investigation not only confirmed a gradation of expression for these genes across patients who achieved pCR, nonpCR with CS, and nonpCR without CS but also aligned with a corresponding gradation in imaging feature changes observed in these groups. This correspondence underlines the credibility of radiomic signatures, such as sphericity, which is indicative of a tumor’s roundness and was found to be associated with a pCR and a CS pattern.

Our genomic analyses confirmed that survival differences among the distinct response pattern groups, as predicted by our model, are underpinned by biological variations. Notably, enrichment of the IL-17 signaling pathway aligns with its known contribution to the invasive progression of breast cancer [34, 35]. This observation is corroborated by evidence showing that IL-1β-induced IL-17 production by γδ T cells drives G-CSF-dependent neutrophil expansion and polarization in breast tumor models, processes critical for disease progression [36]. The complexity of ER signaling has been emphasized [37], with disruptions in ER cofactors and nongenomic mechanisms implicated in the metastasis of ER-positive breast cancer cells [28]. This discovery highlights the critical role of the estrogen pathway in the treatment of hormone-sensitive cancers and in the development of novel drug therapies [38].

Consistent with previous research findings, the extracellular matrix-receptor interaction pathway was notably prominent. This pathway included differentially expressed genes such as those in the THBS family, along with collagen and fibronectin genes, all of which are crucial in breast cancer pathogenesis [39]. These insights provide a valuable understanding of the molecular framework that dictates tumor behavior and the effectiveness of therapeutic interventions.

Our study has several limitations. First, the inclusion of diverse histopathological cancer subtypes, although representative of the clinical spectrum, adds variability to the chemotherapy protocols used. This variability may affect the pCR rate following NAC, potentially introducing selection bias. Second, the use of imaging data from multiple sources with differing imaging protocols may lead to biases in the development of the model, possibly affecting its predictive accuracy and applicability in various clinical settings. To mitigate these issues, future studies should focus on standardizing imaging protocols and taking histopathological variability into account. Such measures would enhance the validation process of the model, ensuring its dependability and usefulness across a wider range of clinical situations.

In summary, our investigation highlights the potential of noninvasive imaging as a prognostic tool for predicting responses to NAC and tumor shrinkage patterns. The study indicated that CS patterns correlate with better survival, particularly in patients who are less likely to reach a pCR. Additionally, gene expression analyses revealed distinct oncogenic pathways associated with various response patterns. These findings support the utility of imaging biomarkers in predicting therapeutic outcomes, emphasizing the role of radiomics in refining early prognosis and enabling personalized therapy.

### Supplementary Information

Supplementary Material 1.

## Data Availability

The gene expression data used in this study were sourced from the Gene Expression Omnibus (GEO) database and identified by the accession numbers GSE32603 and GPL14668. The ISPY-1 trial is available in TCIA on the website: (https://wiki.cancerimagingarchive.net/display/Public/ISPY1). The raw data from development dataset cannot be publicly available but can be obtained upon official request and ethical approval by contacting the corresponding author. PyRadiomics (https://pyradiomics.readthedocs.io/en/latest/) was used for radiomic feature analysis. The microenvironment cell population counter (MCP-counter) R package (http://github.com/ebecht/MCPcounter) was used for cell subpopulation estimation.

## Abbreviations

NAC: Neoadjuvant chemotherapy
pCR: Pathological complete response
AUC: Area under the ROC curve
DCE-MRI: Dynamic contrast-enhanced magnetic resonance imaging
OS: Overall survival
CS: Concentric shrinkage
RFS: Recurrence-free survival
HR: Hazard ratio
