# Supplementary material for: Radiomic analysis reveals diverse prognostic and molecular insights into the response of breast cancer to neoadjuvant chemotherapy: a multicohort study
Source: J Transl Med. 2024 Jul 8;22:637. doi: 10.1186/s12967-024-05487-y (PMC11232151; doi:10.1186/s12967-024-05487-y)
Supplement: Supplementary file 1 — Supplementary Material 1. [file 12967_2024_5487_MOESM1_ESM.docx]

**Tables**

Table S1. Imaging features in the multitask learning model

|  | Features | S0 | SM | SM0 |
| --- | --- | --- | --- | --- |
| Tumor region | Shape | Sphericity  Flatness |  | Elongation |
|  | Texture | Dependence Variance  SDHGLE  Dependence Entropy |  | IDMN  Correlation |
| Parenchyma region | Statistics |  |  | Minimum |
|  | Shape | Sphericity |  |  |
|  | Texture | Contrast  Busyness | MCC  LRLGLE  Busyness | GLNN  Correlation  SDHGLE |

SDHGLE=Small Dependence High Gray Level Emphasis;

MCC=maximal correlation coefficient;

LRLGLE=long run low gray level emphasis;

IDMN=inverse difference moment normalized;

GLNN=Gray Level Non-Uniformity Normalized;

SDHGLE=Small Dependence High Gray Level Emphasis.

Table S2. Univariate survival analysis of the imaging features predicting CS or pCR status

| Model | HR | Beta | z score | p |
| --- | --- | --- | --- | --- |
| Recurrence free survival | | | | |
| Predicted CS | 0.512 (0.281-0.935) | 0.307 | -2.179 | 0.029 |
| Predicted pCR | 0.559 (0.312-1.000) | 0.297 | -1.961 | 0.050 |
| Overall survival | | | | |
| Predicted CS | 0.262 (0.091-0.751) | 0.537 | -2.494 | 0.013 |
| Predicted pCR | 0.482 (0.235-0.988) | 0.366 | -1.994 | 0.046 |

Note: CS=concentric shrinkage; pCR=pathological complete response

Table S3. Univariate analysis of imaging features correlated with CS and pCR status

| Feature | CS | | pCR | |
| --- | --- | --- | --- | --- |
|  | P | t | P | t |
| Tumor |  |  |  |  |
| Sphericity^1^ | <10^-3^ | 10.929 | <10^-3^ | 4.229 |
| Flatness^1^ | <10^-3^ | 7.976 | 0.341 | 0.955 |
| Dependence variance^1^ | 0.481 | 0.707 | 0.870 | -0.164 |
| SAHGLE^1^ | 0.001 | -3.395 | 0.006 | -2.776 |
| Idmn^3^ | 0.133 | -1.508 | 0.007 | -2.758 |
| Dependence entropy^1^ | 0.010 | -2.615 | 0.002 | -3.118 |
| Correlation^3^ | 0.010 | 2.603 | 0.161 | 1.409 |
| Elongation^3^ | <10^-3^ | 4.857 | 0.147 | 1.457 |
| Parenchyma |  |  |  |  |
| MCC^2^ | 0.164 | 1.396 | 0.000 | -4.609 |
| LRLGLE^2^ | 0.157 | 1.421 | 0.009 | 2.683 |
| Sphericity^1^ | <10^-3^ | 4.342 | 0.000 | 5.526 |
| GLNN^3^ | 0.024 | 2.274 | 0.000 | 5.367 |
| Contrast^1^ | 0.807 | 0.245 | 0.002 | 3.163 |
| Busyness^2^ | 0.041 | -2.058 | 0.529 | -0.630 |
| Busyness^1^ | 0.298 | -1.044 | 0.088 | -1.717 |
| SAHGLE^3^ | 0.030 | -2.189 | 0.070 | -1.822 |
| Minimum^3^ | 0.997 | -0.004 | 0.290 | -1.061 |

^1^Imaging feature obtained from precontrast images; ^2^Imaging feature obtained from intermediate postcontrast images; ^3^Imaging feature obtained from subtraction between the intermediate image and the precontrast image;

LRLGLE=long run low gray level emphasis; GLNN=gray level nonuniformity normalized;

SAHGLE=Small area high gray level emphasis; SDHGLE=Small dependence high gray level emphasis;
